# Supplementary material for: Time-series transcriptome analysis identified differentially expressed genes in broiler chicken infected with mixed Eimeria species
Source: Front Genet. 2022 Aug 8;13:886781. doi: 10.3389/fgene.2022.886781 (PMC9393255; doi:10.3389/fgene.2022.886781)
Supplement: Supplementary file 2 [file DataSheet1.ZIP › 4dpi_KEGG.Gsea.1625100298584/KEGG_PENTOSE_AND_GLUCURONATE_INTERCONVERSIONS.html]

Details for gene set KEGG\_PENTOSE\_AND\_GLUCURONATE\_INTERCONVERSIONS[GSEA]

|  || Dataset | TMM\_4dpi\_gct\_format\_4dpi\_gct\_format.Class\_4dpi.cls #PC\_versus\_NC.Class\_4dpi.cls #PC\_versus\_NC\_repos |
| Phenotype | Class\_4dpi.cls#PC\_versus\_NC\_repos |
| Upregulated in class | 0 |
| GeneSet | KEGG\_PENTOSE\_AND\_GLUCURONATE\_INTERCONVERSIONS |
| Enrichment Score (ES) | -0.5627772 |
| Normalized Enrichment Score (NES) | -1.4087715 |
| Nominal p-value | 0.095730916 |
| FDR q-value | 0.3194777 |
| FWER p-Value | 0.9912 |
Table: GSEA Results Summary

  

Fig 1: Enrichment plot: KEGG\_PENTOSE\_AND\_GLUCURONATE\_INTERCONVERSIONS      
 Profile of the Running ES Score & Positions of GeneSet Members on the Rank Ordered List

  

| SYMBOL | TITLE | RANK IN GENE LIST | RANK METRIC SCORE | RUNNING ES | CORE ENRICHMENT || 1 | DHDH | na | 106 | 1.605 | 0.1913 | No |
| 2 | UGP2 | na | 5204 | 0.060 | -0.2259 | No |
| 3 | GUSB | na | 6218 | -0.024 | -0.3074 | No |
| 4 | RPEL1 | na | 8866 | -0.270 | -0.4943 | No |
| 5 | CRYL1 | na | 9537 | -0.354 | -0.5060 | Yes |
| 6 | UGT2A1 | na | 10045 | -0.421 | -0.4958 | Yes |
| 7 | XYLB | na | 10437 | -0.486 | -0.4677 | Yes |
| 8 | UGDH | na | 11579 | -0.858 | -0.4557 | Yes |
| 9 | DCXR | na | 11603 | -0.876 | -0.3484 | Yes |
| 10 | UGT1A1 | na | 11907 | -1.313 | -0.2100 | Yes |
| 11 | AKR1B1 | na | 11968 | -1.752 | 0.0035 | Yes |
Table: GSEA details [plain text format]

  

Fig 2: KEGG\_PENTOSE\_AND\_GLUCURONATE\_INTERCONVERSIONS      
 Blue-Pink O' Gram in the Space of the Analyzed GeneSet

  

Fig 3: KEGG\_PENTOSE\_AND\_GLUCURONATE\_INTERCONVERSIONS: Random ES distribution      
 Gene set null distribution of ES for **KEGG\_PENTOSE\_AND\_GLUCURONATE\_INTERCONVERSIONS**
